# Pennsylvania State Association of Master Horseshoers

**Published:** 1897-10

**Authors:** 


### PENNSYLVANIA STATE ASSOCIATION OF MASTER HORSE-SHOERS.

THE third annual convention was opened at Harrisburg on September 6th, at 10 o'clock, in the hall of the Board of Trade. Dr. James T. McAnulty, of Philadelphia, President, Organizer, and Statistician, presided over the deliberations of the body and introduced City Solicitor Seitz, who delivered an eloquent address of welcome in behalf of Mayor Patterson, who was absent from the city.

President McAnulty made a graceful response to Mr. Seitz's fervent words of greeting. The President also read his annual address to the convention, in which he advocated a closer connection between the local organization and those of the State and nation.

Secretary-Treasurer John C. Smiley, of Philadelphia, read an interesting report, tracing the growth of the Association since its inception, three years ago, and giving a clear exposition of its financial and numerical condition to-day.

The afternoon session was well attended, as all of the forty delegates who enrolled themselves in the morning were present, and in addition a number of outsiders who are interested in the care and protection of the horse. At a late hour in the afternoon officers were elected and the place of next year's convention selected.

In the evening an open meeting was held at the Board of Trade rooms, when Dr. Adams, the well-known Philadelphia veterinarian, delivered a lecture on "The Anatomy of the Horse and the Levelling of the Hoof." Other addresses were made informally by members of the convention.

The old officers have been re-elected with the exception of the first and second vice-presidents, A. J. Shnell, of Scranton, being elected first vice-president, and A. Schappel, of Bethelehem, second vice-president.

